# Preference, Knowledge, and Attitudes of Parents Toward Cognitive Behavioral Therapy for Their Children in Riyadh, Saudi Arabia

**DOI:** 10.3389/fpsyg.2021.725083

**Published:** 2021-12-02

**Authors:** Shuliweeh Alenezi, Ibrahim M. Albawardi, Amirah Aldakhilallah, Ghaliah S. Alnufaei, Rahaf Alshabri, Lama Alhamid, Alanoud Alotaiby, Norah Alharbi

**Affiliations:** ^1^Department of Psychiatry, College of Medicine, King Saud University, Riyadh, Saudi Arabia; ^2^Department of Psychiatry, King Fahad University Hospital, Imam Abdulrahman Bin Faisal University, Dammam, Saudi Arabia

**Keywords:** mental health disorders, parents, cognitive behavioral therapy, children & youth, attitude, preference, knowledge, parents

## Abstract

**Introduction:** Cognitive behavioral therapy (CBT) for children and adolescents has shown efficacy in treating different psychiatric disorders. It has been added to multiple clinical guidelines as the first-line treatment. However, despite more studies of its efficacy, CBT is underutilized in clinical settings due to a lack of rigorous training programs and qualified CBT therapists. The limited knowledge of parents in this intervention and their negative attitudes toward it have been considered as possible reasons.

**Methods:** This is a cross-sectional survey-based study among 464 Saudi parents living in Riyadh city. We aimed to evaluate the preference, knowledge, and attitudes of Saudi parents toward CBT for their children. We compared the difference in the level of knowledge and attitudes toward CBT in relation to the characteristics of parents. An online questionnaire that included 39 questions was carefully reconstructed from four validated scales, approved by an expert panel, and piloted. Participants were recruited to participate through online social media.

**Results:** Saudi parents had average knowledge about CBT; however, they had positive attitudes toward the therapy itself and its role in treating the behavioral issues of children. Male participants showed better knowledge than female participants. Participants with higher education and those with high income had more favorable attitudes toward CBT than others.

**Conclusion:** The knowledge of parents is considered inadequate and indicated the need for more awareness and perhaps mass education. In contrast, they maintained positive attitudes and were interested in evidence-based treatment, with more preference toward non-psychopharmacological interventions.

## Introduction

Cognitive behavioral therapy (CBT) for children and adolescents has shown efficacy in treating different psychiatric disorders. It also has been mounting evidence from different treatment guidelines to be a first-line treatment recommendation for children and youth with many mental illnesses. For instance, in depression, NICE guidelines recommend CBT as a first-line intervention for mild-to-moderate symptoms (Murray and Cartwright-Hatton, 2006; Oud et al., 2019). In anxiety disorder and obsessive-compulsive disorder (OCD), CBT appears to be as effective as medication and has positive results in the tolerance of parents and children for stress related to OCD (James et al., 2013; Selles et al., 2018; Uhre et al., 2020). There are also positive results of CBT efficacy in insomnia and substance use for youth (Hogue et al., 2018; Ma et al., 2018). Although evidence is still growing, CBT has shown efficacy in treatment of early-onset psychosis, migraine, and chronic pain (Ng et al., 2017; Anagnostopoulou et al., 2018; Tang et al., 2018). In addition, in neurodevelopmental disorders, CBT has evidence of efficacy in improving quality of life, functioning, and adaptive functioning in patients with autistic spectrum disorder, as well as for anxiety symptoms associated with the disorder (Sukhodolsky et al., 2013; Ahn and Hwang, 2018; Yang et al., 2019). CBT also has benefits in anxiety and depression symptoms within attention deficit hyperactivity disorder, as well as the symptoms of the disorder itself, primarily through behavioral intervention (Goode et al., 2018; Lambez et al., 2020).

However, the attitudes and expectations of parents toward therapeutic intervention contribute to the responsiveness of children, and those attitudes might influence the quality of therapy, the relationship with the therapist, and the final outcome (Greenberg et al., 2006). Negative expectations, for example, might be a predictive factor of premature termination (Nock and Kazdin, 2001). Recent healthcare developments have emphasized the importance of adequately understanding the perspectives of both patients and parents on treatment. For example, the president of the American Psychological Association has highlighted the regard for the expectations of patients as a critical field of assessment (American Psychological Association, 2005). When treating children, this concern extends to parents who are increasingly encouraged to play an active role in making decisions about the healthcare of their children (Breeding and Baughman, 2003). The effectiveness of care may be positively affected by assessing the preferences of parents. Therefore, according to research with depressed adolescents, treatment outcomes can be enhanced if parents have positive attitudes toward the treatment of their children (Brent et al., 1997). Furthermore, many have concluded that adherence and behavioral improvement increase when parents believe that the therapy is appropriate for their child (Miller and Kelley, 1992).

In Saudi Arabia, the literature showed a suboptimal attitude toward different aspects of psychiatric illnesses. For instance, one study found that 87.5% of the Saudi population has poor knowledge about the nature of psychiatric illnesses, 66.5% have negative attitudes toward mental illness, and 54.5% have negative attitudes toward seeking proficient help (Abolfotouh et al., 2019). Among healthcare providers, one study showed that more than half of the general practitioners and specialists have negative attitudes toward psychiatric patients, and almost half of the general practitioners had never referred patients to the psychiatry department (Al-Atram, 2018). In another study that was conducted on non-psychiatrist physicians, although they were confident in depression management and held positive attitudes toward patients with depression, they stated a preference for dealing with physical rather than mental illness, a lack of confidence in the management of suicidal ideation, and had pessimistic explanations for the cause of depression (Aldahmashi et al., 2019). To our knowledge, there has only been one study that assessed parental attitudes toward the prescription of psychotropic medication to their children, which showed that almost 85% of the participants agreed to give psychotropic medication to their children if necessary, but more than half of the participants had poor knowledge about psychotropic medications (Al-Haidar, 2008).

We initiated the CBT program for children and adolescents in our hospital, i.e., the King Khalid University Hospital (KKUH). However, we faced many challenges in recruiting and maintaining parents and patients to complete the program. Some of those challenges were pertinent to the attitudes of parents and their agreement with and knowledge about CBT. At other times, it was parents favoring psychopharmacological interventions over a long wait list for CBT. Until present, no studies have been conducted in Saudi Arabia that assessed parental knowledge or attitudes toward psychotherapy or CBT for their children and how much they prefer medication over psychotherapy. In this study, we aimed first to measure the preference of Saudi parents for medication and/or psychotherapy for their children. Then, we would like to further assess their fund of knowledge, and finally, their attitudes toward CBT, which were divided into three factors, namely, perceived usefulness, responsibility, and effectiveness, as a possible intervention to consider for their children when indicated.

## Materials and Methods

### Participants and Study Design

This cross-sectional study was part of the King Saud University Cognitive Behavioral Therapy Program for children with anxiety (KSU-CBT). The data were collected from September 2020 to October 2020. As the most recent report of the Saudi Communication and Information Technology Commission stated that more than 91% of the Saudi population uses the Internet, with more than 87% and more than 55% of the Saudi population using WhatsApp and Twitter, respectively, and due to the precautions of coronavirus disease 2019 (COVID-19) during data collection, we used an online questionnaire to collect the data. Participants were able to access the questionnaire through the link that was distributed on Twitter and WhatsApp.

We created an account on Twitter, and tweets were sent directly to both individuals and organizations as a request to retweet the survey link. We also shared the questionnaire link through WhatsApp groups and encouraged people to share the link. The participants understood the objective of the study and provided informed consent. Considering our hospital location and the majority of our patients being Saudis, we restricted our sample to Saudi parents who live in Riyadh and have children between the ages of 7 and 18. Noncitizens, parents who work in the mental health field, and individuals with previous experience with CBT were all excluded, as we assume those will not reflect the actual knowledge of the general population. The age range of children was chosen based on the most common age accepted in CBT programs in Saudi Arabia.

### Measures and Outcomes

Since there is no validated Arabic scale to help answer our research questions, the survey contents were first identified from four different scales in English (Pierce and Pearce, 2003; Donovan et al., 2015; Berg et al., 2019; Kuckertz et al., 2020). Questions were reorganized by the research team, underwent one-way translation into Arabic, and were then reviewed by a panel consisting of a child psychiatrist, child psychologist, and adult psychiatrist. Following this step, it was piloted on 20 parents for appropriateness, comprehension, and accounting for cultural appropriateness; some items were slightly modified as a result. Each item consists of a 5-point Likert scale ranging from 1 (strongly disagree) to 5 (strongly agree). Knowledge questions were divided into yes/no and multiple-choice questions to help assess the main facts about CBT. One of the questions concerning the knowledge of parents about CBT was taken from a study by Berg et al. (2019). We introduced this question to assess whether parents knew that participating in CBT exercises is a prerequisite in such treatments. The survey was entirely in Arabic and took <10 min to complete.

The final survey consisted of 39 questions (Appendix A1) and was divided into six parts:

1. Sociodemographic data: This covered the primary demographic data and included other questions, such as the number of children and previous experience with child CBT.
2. Familiarity with CBT (questions 14–19): We selected questions from the study by Donovan et al. (2015), which we found helpful in our study. We also added the last two questions from the study by Kuckertz et al. (2020).
3. and 4. Knowledge about CBT and Aims and Values of CBT (questions 20–31): These questions were taken from the previous study by Berg et al. (2019). We have selected the questions related to the principles presented in CBT. We also assessed the knowledge about CBT and the aims and values of CBT using the same questions used in the study by Pierce and Pearce (2003).
5. Attitude toward CBT (questions 32–37): We used the Psychological Treatment Consumer Questionnaire (PTCQ) (Kuckertz et al., 2020). We have modified the Familiarity section with specific evidence-based psychological treatments to be suitable for CBT typically offered to children.
6. Agreement with CBT (questions 38–39): We used the same questions that were used by Donovan et al. (2015).

After data collection and before analysis, the research team, in consultation with the team biostatistician, reorganized survey questions to five different themes to provide the survey with more consistency and control after being reconstructed from four different scales to meet our research objectives. After redistribution, we identified the following five themes: knowledge about CBT, perceived general usefulness of CBT, perceived CBT responsibility, perceived trust/effectiveness of CBT, and the overall attitude toward CBT. We then analyzed the data accordingly (Appendix A2).

In regard to the scoring system, participants were asked to rate the answers on a 5-point Likert scale (1 = strongly disagree, 2 = disagree, 3 = undecided or neutral, 4 = agree, and 5 = strongly agree). Additionally, in assessing the preference of parents for the mental health treatments of their children, the five options provided were scored as follows: 1 = A combination of medication and psychotherapy, 2 = Do not prefer psychotherapy at all, 3 = Medication only, 4 = Psychotherapy only, and 5 = Peer support group only. Furthermore, parents were asked to indicate, with regard to decision-making when it comes to the mental health treatment of their children, whether they would decide based on the treatment recommendation of providers or would prefer treatment that research suggests is most effective for their child. Some questions were rated on a 3-point Likert scale (1 = disagree, 2 = undecided, and 3 = agree). The options for a few questions were yes, somewhat, or no, and the answers were rated as 1, 2, and 3, respectively. For the knowledge section, each correct answer was given a score of 1, and each wrong answer was given a score of 0. Finally, for yes/no questions, they were given a score of 1 = yes and 0 = no. The minimum and maximum scores for each section are presented in **Table 3**.

The sample size was calculated by the calculator.net website and confirmed manually by the following equation: *n* = *z*^2^*p* (1 − *p*)/*d*^2^, with a proportion of 50% of parents having good knowledge and positive attitudes, *z* = 1.96 (95% CI), and *d* = 5% (margin of error). The estimated sample size is 385 participants, and an additional 20% was added to the original sample size to anticipate nonresponse participants. Out of 582 responses in total, 464 were included, and the main reasons for exclusion were as follows: noncitizens, parents who work in the mental health field, and individuals with previous experience with CBT.

### Statistical Methods

The data were analyzed using SPSS version 24.0 statistical software. Descriptive statistics (mean, SD, frequencies, and percentages) were used to describe the quantitative and categorical variables. The bivariate statistical analysis was carried out using appropriate (Student's *t*-test and one-way ANOVA) statistical tests for the quantitative outcome variables. A *p* < 0.05 reports the statistical significance of the results. The informed consent was clear and indicated the purpose of the study and the right of the participant to withdraw at any time without any obligation toward the study team. Participant anonymity was assured by assigning each participant a code number for the purpose of analysis only.

## Results

In this study, 464 Saudi parents electively enrolled themselves and completed the survey. The sociodemographic characteristics of respondents are shown in Table 1. The Cronbach's alpha test of reliability suggested that the nine items measuring the attitude of the people toward CBT were found to be reliable, Cronbach's α = 0.74, suggesting that people had reliably read and understood these items.

**Table 1:** Distribution of the sociodemographic characteristics of parents (*N* = 464).

	**No**	**(%)**	**Mean (SD)**
**Sex**			
Female	381	82.1	
Male	83	17.9	
**Age (years)-**			43 (8.7)
**Age groups**			
20–30 years	33	7.1	
31–40 years	169	36.4	
41–50 years	177	38.1	
>50 years	85	18.3	
**Marital status**			
Widowed	16	3.4	
Divorced	25	5.4	
Married	423	91.2	
**Educational level**			
Elementary	6	1.3	
Intermediate	12	2.6	
Secondary	80	17.2	
Diploma	45	9.7	
University degree	274	59.1	
Master's degree	32	6.9	
PhD	15	3.2	
**Employment state**			
Unemployed	200	43.1	
Employed	264	56.9	
**Household monthly income (SAR)**			
<5,000	80	17.2	
5,000–10,000	107	23.1	
11,000–15,000	139	30.0	
>15,000	138	29.7	

**Figure 1:**
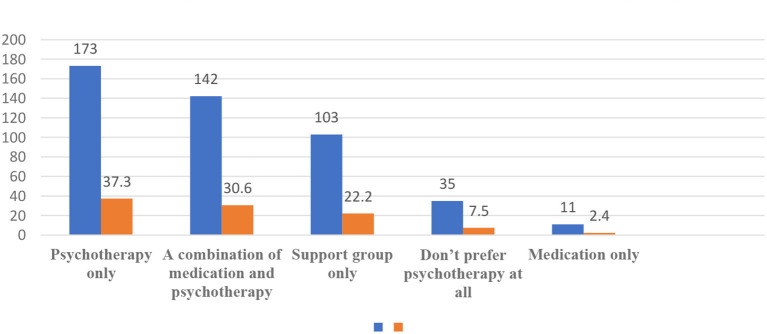
The preference of parents toward modality of treatment for their children (*N* = 464).

To assess the preferred treatment modality of respondents to the behavioral problems of children, they were asked to indicate their intervention of choice from five different options. The findings showed that 7.5% of the respondents did not prefer psychotherapy at all, and another 2.4% preferred medications only. Nevertheless, another 22.2% of respondents preferred support groups, and most parents, 37.3%, preferred only psychotherapy, with the remaining 30.6%, preferring a combination of medication and psychotherapy (Figure 1).

**Figure 2:**
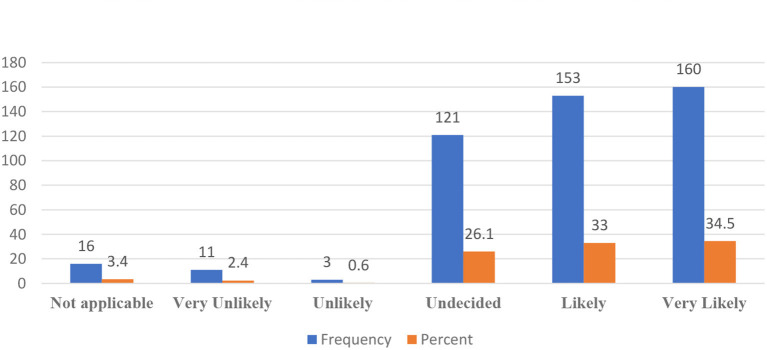
Likelihood to recommend cognitive behavioral therapy (CBT) to a friend with a child having emotional difficulties.

**Figure 3:**
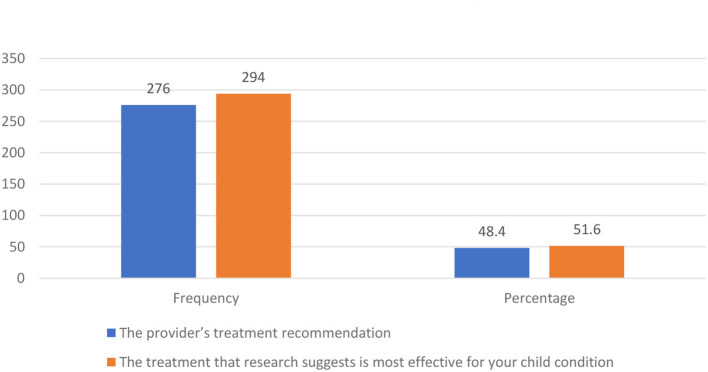
Main influence on treatment decision of parents.

Based on what parents knew about CBT, our findings showed that 34.5% of respondents were very likely to recommend it to a friend with a child with emotional difficulties, with an additional 33% being likely to do so. However, 26.1% of the total respondents had not yet made up their minds about whether or not they would recommend it, and 3.4% do not see themselves as applicable to such conditions. Finally, 2.4 and 0.6% of the respondents feel that it was very unlikely and unlikely, respectively, that they would recommend CBT to a friend with a child facing emotional difficulties (Figure 2). When it comes to making decisions about the mental health treatment of their children, 51.6% of the respondents preferred treatment that research suggested as most effective for the condition of their children. In contrast, 48.4% of respondents preferred the treatment recommendation of providers (Figure 3).

Table 2 displays the yielded data analysis of the measured knowledge of parents on CBT-specific facts. Most of the respondents, 87.3%, had correctly inferred that the mood of a person is a result of their actions and a consequence of their behaviors. Still, 68.5% of respondents had incorrectly inferred that according to CBT, the thoughts of someone have an impact on their mood and that the best mood-changing action is thinking positive thoughts rather than negative ones; another 12.3% of the respondents misbelieved that ignoring thoughts could be the best action to control mood. Only 19.2% of the respondents correctly inferred that learning to recognize what is on the mind of someone before building a specific feeling is part of CBT. In addition, 33 of the respondents correctly inferred that it is essential to be active and do the exercises as part of CBT because it is a prerequisite for participating in such treatment. However, 57.1% of respondents incorrectly inferred that such exercises and active participation were meant to assimilate new skills, which is a partially correct answer, as it does not recognize that agreeing to participate in CBT homework is a prerequisite to join any CBT program. Nevertheless, 9.9% of respondents incorrectly inferred that such exercises and active participation were not required because it can be so demanding for someone who is depressed to participate in such therapy. Of note, 64.4% of the participants correctly inferred the primary focus of CBT to be working out what is currently and actively problematic, 13.6% incorrectly believed that the core aim of CBT was working out thoughts about the future, and another 22% believed that CBT focuses on past events and previously encountered issues.

**Table 2:** The parental knowledge about cognitive behavioral therapy (CBT) (*N* = 464).

	**Frequency**	**Percentage (%)**
**According to CBT, the mood is a result of what we do and the consequences of those behaviors?**		
False	59	12.7
True^**^	405	87.3
**According to CBT, our thoughts have an important impact on our mood. What is important to do if you are trying to change your mood?**		
Learn to recognize what is on your mind since you almost always think something before a certain feeling^**^	89	19.2
Try to ignore your thoughts and do not waste any energy on them	57	12.3
Think more positive thoughts than negative thoughts	318	68.5
**Is it important to be active and do the exercises included in CBT?**		
No, if you feel depressed it can be too demanding, which makes you feel even worse	46	9.9
Yes, to assimilate new skills, you need to practice them actively	265	57.1
Yes, it is a prerequisite to participate in such treatments^**^	153	33
**What is the primary focus in a CBT treatment?**		
To work with previous events and issues	102	22
To work with what is problematic here and now^**^	299	64.4
To work with thoughts about the future	63	13.6

** *Best correct answers*.

The knowledge of parents about CBT was measured at 2.04 out of 4 maximum points, which is equivalent to 2.04/4 × 100 = 51% knowledge. In addition, the perceived usefulness of CBT was measured with 12.73 out of 15 points, which is equivalent to 84.86% agreement on the usefulness of CBT for treating the behavioral problems of children. Furthermore, the attitudes of people toward the responsibility of learning CBT were measured with 12.60 points out of 15 points, which highlights an overall high agreement by those respondents on the importance of learning the concepts of CBT and its components. Nonetheless, the perceived effectiveness of CBT in treating the mental issues of children was rated with 9.18 out of 11 points, or 83.45% perceived effectiveness. The overall attitudes of respondents toward CBT, which were comprised of their perceived usefulness, responsibility, and effectiveness of CBT, were rated 34.16 out of 41 points. This overall attitude score highlights great perceived attitudes by the respondents toward the CBT usefulness, responsibility, and effectiveness combined. In short, respondents had average knowledge about CBT, but they had great attitudes toward the therapy itself and its role in treating the behavioral issues of children (Table 3).

**Table 3:** Descriptive statistics for the measured knowledge and attitudes of respondents of the people toward CBT (*N* = 464).

	**Mean (SD)**	**Minimum and maximum score**
Knowledge score about CBT	2.04 (0.82)	0–4 points
Perceived CBT general usefulness	12.37 (1.67)	3–15 points
Perceived CBT responsibility	12.60 (1.75)	3–9 points
Perceived trust/effectiveness of CBT	9.18 (1.63)	3–11 points
Overall attitude toward CBT	34.16 (3.68)	11–41 points

To better understand the knowledge and attitudes of parents toward CBT, differences across sociodemographic factors were analyzed. The results of the findings (Table 4) suggested that differences in the educational levels, household monthly income, marital status, and employment status of respondents did not factor significantly into their knowledge of CBT. However, the *t*-test showed that the mean knowledge of CBT differed significantly between genders. In contrast, the results of the findings suggested that the differences in the gender, age, marital status, and employment status of respondents did not factor significantly into their respective mean attitude scores (Table 5). However, a one-way ANOVA test suggested that the educational levels of parents might differ in their mean perceived attitudes toward CBT, *F*_(6, 457)_ = 2.918, *p* < 0.08 Furthermore, the household monthly income of respondents showed a statistically significant difference, with people with a monthly income >15,000 Saudi Riyals having the highest scores.

**Table 4:** Comparison of mean values of the knowledge of respondents of parents about CBT in relation to their personal characteristics (*N* = 464).

	**Mean (SD)**	**Test statistic**	***p*-value**
**Sex**			
Female	1.98 (0.796)	*t*_(112.094)_ = −3.009^*^	0.003
Male	2.30 (0.893)		
**Employment status**			
Unemployed	2.01 (0.760)	*t*_(452.262)_ = −0.784^*^	0.433
Employed	2.06 (0.867)		
**Age group**			
20–30 years	2.27 (1.008)	*F*_(3, 460)_ = 1.389^**^	0.245
31–40 years	1.98 (0.798)		
41–50 years	2.02 (0.780)		
>50 years	2.11 (0.873)		
**Marital status**			
Widowed	1.88 (0.719)	*F*_(2, 461)_ = 0.468^**^	0.627
Divorced	1.96 (0.676)		
Married	2.0496 (0.835)		
**Educational level**			
Elementary	2.50 (0.548)	*F*_(6, 457)_ = 1.779^**^	0.102
Intermediate	1.75 (0.754)		
Secondary	1.96 (0.754)		
Diploma	2.16 (0.638)		
University degree	2.02 (0.847)		
Master's degree	2.00 (0.950)		
PhD	2.53 (0.915)		
**Household monthly income (SAR)**			
<5,000 SAR	1.99 (0.665)	*F*_(3, 460)_ = 0.894^**^	0.444
5,000–10,000 SAR	1.96 (0.889)		
11,000–15,000 SAR	2.04 (0.797)		
>15,000 SAR	2.12 (0.875)		

* *By the Student's t-test for two independent variables*;

** *By one-way ANOVA*.

**Table 5:** Comparison of mean values of the attitude of respondents of parents toward CBT in relation to their personal characteristics (*N* = 464).

	**Mean (SD)**	**Test statistic**	***p*-value**
**Sex**			
Female	34.12 (3.60)	*t*_(112.40)_ = −0.37^*^	0.71
Male	34.30 (4.02)		
**Age group**			
20–30 years	35.18 (3.27)	*F*_(3, 460)_ = 1.173^**^	0.32
31–40 years	34.21 (3.77)		
41–50 years	34.08 (3.41)		
>50 years	33.78 (4.13)		
**Marital status**			
Widowed	34.31 (4.22)	*F*_(2, 461)_ = 0.050^**^	0.951
Divorced	33.96 (3.55)		
Married	34.16 (3.67)		
**Educational level**			
Elementary	32.50 (1.76)	*F*_(6, 457)_ = 2.917^**^	0.008
Intermediate	32.58 (5.16)		
Secondary	33.50 (3.77)		
Diploma	32.93 (2.99)		
University degree	34.56 (3.67)		
Master's degree	35.18 (3.28)		
PhD	33.60 (3.68)		
**Employment status**			
Unemployed	34.23 (3.84)	*t*_(462)_ = 0.38^*^	0.704
Employed	34.09 (3.55)		
**Household monthly income (SAR)**			
<5,000 SAR	32.91 (4.01)	*F*_(3, 460)_ = 7.43^**^	0.00
5,000–10,000 SAR	33.67 (3.77)		
11,000–15,000 SAR	34.23 (3.21)		
>15,000 SAR	35.16 (3.60)		

* *By the Student's t-test for two independent samples*;

** *By one-way ANOVA*.

## Discussion

The purpose of this study was to investigate the preference of Saudi parents for psychotherapy and/or medication and their basic knowledge and attitudes in considering CBT for their children. During the assessment of the preference of parents toward the modality of treatment for their children, we found that the majority of parents prefer either psychotherapy alone or a combination of psychotherapy and pharmacological interventions. These findings are consistent with the study conducted by Brown et al. (2007), which affirmed that CBT was generally perceived as acceptable, believable, and effective. In the same study, parents similarly perceived CBT to be more acceptable and favorable than medication. The rest of our sample was split into preferring support groups and did not prefer psychotherapy at all, and a small fraction preferred only pharmacological therapy. We consider this a surprising finding based on our experience in recruiting and maintaining parents to join our CBT program, as the majority of families did not complete the program. However, this was in agreement with other studies, which found that CBT is typically more accepted by parents than medication (Miller and Kelley, 1992). In addition, other studies on treatment expectations and preferences of parents of children with different internalizing disorders have shown that parents prefer CBT over medication (Dudley et al., 2005).

Parents are encouraged to ask their providers for further information about psychotherapy; it is also crucial that clinicians understand how to explain and offer these interventions properly. In line with this view, half of the parents (50.2%) stated that it was a joint responsibility of themselves and their providers to provide information about treatment options. Moreover, half of the respondents (49.8%) said that the recommendation of their providers is more important in treatment selections than research findings. Those results were lower than those published in the study by Kuckertz et al. (2020) (58%). These data suggest that patients have expectations of their healthcare providers in terms of treatment education. It may, however, necessitate a reevaluation of the connection between parents and healthcare practitioners in our community.

The CBT, especially for children, is considered a new modality in the Saudi culture with a few number of therapists who could provide this intervention. Thus, assessing the general knowledge of parents about CBT was considered an important aspect of our study. In our results, we found that the majority of participants were able to understand the relationship between mood and behavior; however, and one-third knew the importance of active participation in the therapy. While the best outcome in CBT is achieved through combining behavioral and cognitive intervention (Oud et al., 2019), the impact of behavior on mood was more understood by parents than the impact of thoughts on mood. Still, this might explain how CBT is easy to understand and considered an important option for parents (Algahtani et al., 2017). In contrast, realizing this potential knowledge gap among parents could help CBT program designers describe how thoughts, moods, and behaviors interact based on the CBT model. It might also indicate that CBT is underutilized in clinical settings in Saudi Arabia, a finding reported worldwide and thought to be related to a scarcity of rigorous training programs and qualified mental health professionals of CBT (Myhr and Payne, 2016). In addition, half of the parents did not recognize the importance of being active in participating in CBT exercises, which is a mandate to participate in CBT. This was very concerning and might reflect the difficulty in retaining families who agreed to join our local program. The importance of parental participation in CBT has been documented, as this aids and strengthens the good learning, maintenance, and generalization of new skills and experiences of the children and family in everyday life, both during and after treatment (Stallard, 2005). We believe that this might relate to parents treating CBT as a generic skill-learning activity and not being aware of the regular exercise and homework commitments.

Interestingly, no reports that discussed the link between parental education or level of income and preference to psychotherapy were found. Still, our sample found an association, which was more prominent, that parents with higher education and higher income had positive attitudes toward CBT. This finding could be explained by the fact that CBT is still considered a relatively new modality in Saudi Arabia, and most likely, parents who were more educated and wealthier were aware of this critical intervention. In addition, the limited number of therapists in Saudi Arabia and the high therapy fees make it more accessible to parents with higher incomes. As a result, there have been attempts to fill the gap by training more therapists to fill this likely unmet need for this therapeutic approach (Beck et al., 2016). However, we think that this will continue as a healthcare gap, as training therapists to administer this intervention has traditionally been a time-consuming and costly procedure, with already highly trained personnel such as psychiatrists and psychologists undergoing further training lasting up to a year to develop expertise in this field (Beck et al., 2016). Recognizing this shortcoming, a core group of Western-trained Saudi psychiatrists and psychologists certified in CBT are leading the scene by organizing workshops for practitioners on the fundamentals of CBT and specific illness-specific CBT methods. Hence, psychiatry residency programs are now mandated to provide psychotherapy, and more recently, training programs must support trainees in demonstrating working knowledge in at least one of the following: interpersonal psychotherapy, CBT, psychodynamic therapy, family therapy, group therapy, and/or supportive therapy (Saudi Council for Health Specialities, 2016).

As this study addresses parents choosing CBT for their children, findings were not different from other reports about the influence of gender on psychotherapy preference, particularly CBT. For example, the Sequenced Treatment Alternatives to Relieve Depression Study (STAR^*^D) did not find a gender preference for CBT or any gender differences in response to CBT compared with medication (Thase et al., 2007). However, we found that males were more knowledgeable about CBT compared to females. Although it was challenging to explain gender differences in the knowledge gap, several meta-analyses of psychotherapy outcomes excluded the analysis of gender as potential variables, resulting in the limitations of the literature (Weissman, 2014). Nevertheless, the lack of research on the preferences and sociodemographic characteristics of parents, which might influence this decision, will make it difficult to assess this area or infer valuable associations.

## Conclusion/Recommendations

This study aimed to measure treatment preference, level of knowledge, and attitudes of Saudi parents toward CBT for their children. Based on the analysis, parents have a more positive attitude toward CBT than hypothesized, but their knowledge was found to be more limited than their attitudes. These results might encourage more efforts to fulfill a higher level of knowledge about CBT among Saudi parents by utilizing the newly established Ministry of Health Community Empowerment Initiative. Further studies are needed to assess the knowledge and attitudes of the population toward CBT in Saudi Arabia and to consider validating a scale for that purpose.

## Limitations

The self-reported data increased the possibility of non-response and recall bias. The questionnaire was not validated and had long questions that may have impacted the response rate and the accuracy of the reported data. The limited sample to one city in Saudi Arabia and the significant gender variation in our study might limit the generalizability of the study. The survey questions had limited consistency and high heterogeneity, so more elaborating questions are needed to increase reliability, especially for the knowledge section.

## Data Availability Statement

The raw data supporting the conclusions of this article will be made available by the authors, without undue reservation.

## Ethics Statement

The studies involving human participants were reviewed and approved by Institutional Review Board at King Saud University College of Medicine. The patients/participants provided their written informed consent to participate in this study.

## Author Contributions

SA, IA, AAld, and GA conceived the study and designed the research methods. NA, LA, AAld, RA, and AAlo collected the research data. AAld prepared the data analysis plan. LA, IA, GA, and RA wrote the manuscript under the guidance of SA. All authors contributed to the article and approved the submitted version.

## Conflict of Interest

The authors declare that the research was conducted in the absence of any commercial or financial relationships that could be construed as a potential conflict of interest.

## Publisher's Note

All claims expressed in this article are solely those of the authors and do not necessarily represent those of their affiliated organizations, or those of the publisher, the editors and the reviewers. Any product that may be evaluated in this article, or claim that may be made by its manufacturer, is not guaranteed or endorsed by the publisher.
