# Supplementary material for: Preference, Knowledge, and Attitudes of Parents Toward Cognitive Behavioral Therapy for Their Children in Riyadh, Saudi Arabia
Source: Front Psychol. 2021 Dec 2;12:725083. doi: 10.3389/fpsyg.2021.725083 (PMC8675882; doi:10.3389/fpsyg.2021.725083)
Supplement: Supplementary file 1 [file Data_Sheet_1.docx]

Appendix A

Table A1: Survey used in English

| **Q no.** |  |
| --- | --- |
| **1** | **Sex** |
|  | Female |
|  | Male |
| **2** | **Age groups** |
|  | 20–30 years |
|  | 31–40 years |
|  | 41–50 years |
|  | >50 years |
| **3** | **Nationality** |
|  | Non-Saudi |
|  | Saudi |
| **4** | **Marital state** |
|  | Widowed |
|  | Divorced |
|  | Married |
| **5** | **Residence** |
|  | Other places |
|  | Central region |
| **6** | **Educational Level** |
|  | High school or less education |
|  | Diploma Level |
|  | University Degree |
|  | Higher studies |
| **7** | **Employment state** |
|  | Unemployed |
|  | Employed |
| **8** | **Household monthly income (SAR)** |
|  | <5000 SAR |
|  | 5000–10000 SAR |
|  | 11000–15000 SAR |
|  | >15000 SAR |
| **9** | **Do you work in the field of psychology?** |
|  | No |
|  | Yes |
| **10** | **Any relatives working in the field of psychology?** |
|  | None |
|  | One of the parents |
|  | A Relative |
| **11** | **How many children do you have?** |
| **12** | **Do you have a child aged between 7–18 years?** |
|  | No |
|  | Yes |
| **13** | **If you answered the previous question with yes, how old is/are he/they ? (you can choose more than 1 answer)** |
|  | 7 |
|  | 8 |
|  | 9 |
|  | 10 |
|  | 11 |
|  | 12 |
|  | 13 |
|  | 14 |
|  | 15 |
|  | 16 |
|  | 17 |
|  | 18 |
| **14** | **Have you had ever seen CBT program in use?** |
|  | No |
|  | Yes |
| **15** | **Have you had ever been personally involved in any CBT program?** |
|  | No |
|  | Yes |
| **16** | **Did any of your children require CBT?** |
|  | No |
|  | Yes |
|  | ***Parents’ preference to treatment modality*** |
| **17** | **How effective you felt CBT was in treating your child if your child was treated with CBT:** |
|  | Not at all effective |
|  | Very Little effect |
|  | A little Impact |
|  | Somewhat effective/quite effective |
|  | Effective |
|  | Wasn't treated before |
| **18** | **Based on what you’ve heard or know about it, what is your overall impression of cognitive behavioral therapy (CBT) in children?** |
|  | Never experienced CBT |
|  | Negative |
|  | Neutral |
|  | Positive |
| **19** | **Based on what you’ve heard or know about it, how likely is it that you would recommend group cognitive behavioral therapy (CBT) in children to a friend with children with emotional difficulties?** |
|  | Not applicable  Very Unlikely  Unlikely  Undecided  Likely  Very Likely |
| **20** | **What is your current preference for your child’s mental health treatment?** |
|  | A combination of medication and psychotherapy  I don’t prefer psychotherapy at all  Meds only  Psychotherapy only  Support group only |
| **21** | **When it comes to making decisions about your child mental health treatment, which is more important to you?** |
|  | The provider’s treatment recommendation. |
|  | The treatment that research suggests is most effective for your child condition |
|  | ***Knowledge**** |
| **22** | **According to CBT, the mood is a result of what we do and the consequences of those behavior.** |
|  | False |
|  | True** |
| **23** | **How confidant are you about your answer ?** |
|  | I'm guessing |
|  | Somehow confident |
|  | I'm confident |
| **24** | **According to CBT, our thoughts have an important impact on our mood. What is important to do if you are trying to change your mood?** |
|  | Learn to recognize what is on your mind since you almost always think something before a certain feeling.** |
|  | Try to ignore your thoughts and do not waste any energy on them |
|  | Think more positive thoughts than negative thoughts |
| **25** | **How confidant are you about your answer ?** |
|  | I'm guessing |
|  | Somehow confident |
|  | I'm confident |
| **26** | **Is it important to be active and do the exercises included in CBT?** |
|  | No, if you feel depressed it can be a too demanding, which makes you feel even worse |
|  | Yes, in order to assimilate new skills, you need to practice them actively |
|  | Yes, it is a prerequisite in order to participate in such treatments** |
| **27** | **How confidant are you about your answer ?** |
|  | I'm guessing |
|  | Somehow confident |
|  | I'm confident |
| **28** | **What is the primary focus in a CBT treatment?** |
|  | To work with previous events and issues |
|  | To work with what is problematic here and now** |
|  | To work with thoughts about the future. |
| **29** | **How confidant are you about your answer ?** |
|  | I'm guessing |
|  | Somehow confident |
|  | I'm confident |
|  | ***Overall attitudes questions*** |
| **30** | CBT allows patients to recognize and deal with maladaptive thinking we do and the consequences of those behaviors. |
| **31** | CBT aims to change behavior that perpetuates symptoms. * |
| **32** | CBT is a useful tool in general practice. * |
| **33** | I feel CBT techniques are helpful in my personal life. * |
| **34** | It is important to you that your child receives psychotherapy that is supported by research* |
| **35** | It is important to be informed about different options for psychotherapy* |
| **36** | It is my responsibility to make sure I am aware of different options for psychotherapy.* |
| **37** | It is the provider’s responsibility to inform me about different options for psychotherapy* |
| **38** | Do you agree with the principles of CBT?** |
| **39** | Do you believe CBT is an effective therapeutic approach? ** |
|  | * 1-5 Likert scale questions  ** Indicate right answers |
|  |  |

| **Table A2: Themes of parents' indicators of knowledge and attitudes toward CBT.** | | | |  |  |
| --- | --- | --- | --- | --- | --- |
| **Questions used**  **for each theme** | **Components** | | |  |  |
|  | **Perceived CBT usefulness** | **Responsibility** | **Trust in CBT/effectiveness** | **Knowledge** | **Overall attitude toward CBT** |
| CBT is a useful tool in general practice. | * |  |  |  | * |
| I feel CBT techniques are helpful in my personal life. | * |  |  |  | * |
| CBT aims to change behavior that perpetuates symptoms. | * |  |  |  | * |
| It is the provider’s responsibility to inform me about different options for psychotherapy. |  | * |  |  | * |
| It is important to be informed about different options for psychotherapy. |  | * |  |  | * |
| It is my responsibility to make sure I am aware of different options for psychotherapy. |  | * |  |  | * |
| Do you agree with the principles of CBT? |  |  | * |  | * |
| Do you believe CBT is an effective therapeutic approach? |  |  | * |  |  |
| What is your current preference for your child mental health treatment? |  |  | * |  |  |
| According to CBT, our thoughts have an important impact on our mood. What is important to do if you are trying to change your mood? |  |  |  | * |  |
| According to CBT, the mood is a result of what we do and the consequences of those behaviors. |  |  |  | * |  |
| Is it important to be active and do the exercises included in CBT? |  |  |  | * |  |
| What is the primary focus in CBT treatment? |  |  |  | * |  |
| CBT allows patients to recognize and deal with maladaptive thinking: what we do and the consequences of those behaviors. |  |  |  |  | * |
| It is important to you that your child receives psychotherapy that is supported by research. |  |  |  |  | * |
